# Perceived discrimination and favourable regard toward underweight, normal weight and obese eating disorder sufferers: implications for obesity and eating disorder population health campaigns

**DOI:** 10.1186/s40608-014-0032-2

**Published:** 2015-02-07

**Authors:** Anita Star, Phillipa Hay, Frances Quirk, Jonathan Mond

**Affiliations:** Private Practice, Albury, 2640 New South Wales Australia; School of Medicine and Dentistry, James Cook University, Townsville, Queensland 4811 Australia; School of Medicine, University of Western Sydney, Locked bag 1797, Penrith, New South Wales 2751 Australia; School of Medicine, Deakin University, Melbourne, 3125 Victoria Australia; Research Directorate, Barwon Health, Geelong, 3220 Victoria Australia; Department of Psychology, Macquarie University, Sydney, New South Wales 2129 Australia

**Keywords:** Obesity, Binge-eating, Anorexia nervosa, Body-image, Stigma

## Abstract

**Background:**

Obesity stigma has been shown to increase binge eating, whilst positive regard for eating disorders (EDs) may increase dietary restriction which can also lead to binge eating and weight gain. In the context of increasing prevalence of both obesity and EDs exploring community attitudes towards these illnesses may uncover new variables worthy of consideration in population health campaigns. The aim of the study was to explore community perceived stigma and conversely favourable regard toward eating disorder (ED) sufferers of varying weight status, and understand how the attitudes of obese individuals may differ from those of non-obese individuals. Data for this purpose were derived from interviews with individuals participating in a general population health survey. Vignettes of an underweight female with Anorexia Nervosa (AN), a normal weight male with an atypical eating disorder (NWED) and an obese female with Binge Eating Disorder (BED) were presented to three randomly selected sub-samples of n = 983, 1033 and 1030 respectively. Questions followed that assessed participants’ attitudes towards and beliefs about the person described in the vignette and their eating behaviours.

**Results:**

Sixty-six per cent of participants who responded to the obese BED vignette believed that there would be discrimination against the person described (primarily because of her weight). Corresponding figures were for the AN and NWED vignettes were 48% and 35%, respectively. A positive regard for weight-loss or body-image-enhancing ED behaviours was reported ‘occasionally’ or more often by 8.8% of respondents to the AN vignette and by 27.5% of respondents to the NWED vignette. Positive regard for ED behaviours was significantly more likely in obese participants (AN: 15%; NWED: 43%).

**Conclusion:**

The findings support integrated ED and obesity prevention programs that address weight stigma and the social desirability of ED behaviours in vulnerable individuals.

## Background

In population studies, co-morbidity of disordered eating and obesity is high and increasing, currently affecting approximately 1 in 5 obese persons in the community [1,2]. Eating disorder (ED) psychopathology affecting obese persons includes binge eating, extreme weight control behaviours such as fasting, self-induced vomiting, and laxative misuse, as well as high levels of body image distress and eating concerns [1,2]. Despite the desire to manage body weight and shape, individuals suffering from bulimic eating disorders are at increased risk of weight gain and obesity onset [3-5]. Thus the prevention of eating disorders may offer new hope in the prevention of obesity.

Understanding the factors that might account for the increasing proportion of the population developing comorbid eating disorder behaviours and obesity could provide helpful targets in innovative public health campaigns. To date this possibility has not been explored in representative population-based samples. However, research on stigma and discrimination toward obesity or ED sufferers may hold important clues. Stigma is defined as “a set of negative and often unfair beliefs that a society or group of people have about something” [6]^.^ In this case, the “something” is a person who is obese or who is suffering from an ED. Stigma can result in exclusion and discrimination and cause significant distress. Research has found strong links between the experience of obesity stigma and depression, poor body image and binge eating [7-15]. As body dissatisfaction and negative affect are key risk factors in the development of EDs [16-19], stigma could be an important target in ED prevention. Further, findings from qualitative research suggest that many obese individuals believe that there is an emerging culture of blame and stigma against them which is heightened by the simplicity of public health messages employed in obesity prevention campaigns [20,21]. For example, public health messages may imply that it is easy to lose weight by changing eating and exercise habits which is contrary to many people’s experiences [20,21]. There is also evidence of community stigma towards individuals with EDs [22-29], which has contributed to low rates of help-seeking among sufferers [30-32]. However there have also been reports of positive attitudes towards ED behaviour, particularly the weight-loss and weight-control aspects of this behaviour [24,28,29].

Considerable research has addressed public attitudes toward anorexia nervosa (AN) and bulimia nervosa (BN). Research in the UK [22] and the USA [23] suggests high levels of ED-related stigma, with many people reporting difficulty talking to and empathising with people with EDs, and a general perception that EDs are ‘self-inflicted’, or that sufferers ‘have only themselves to blame’ and ‘could pull themselves together’. Studies in a number of high school and universities have also found stigmatising attitudes toward EDs, with students perceiving ED sufferers to be self-centred, fragile, narcissistic, a danger to others, and likely to use their disorder to gain attention. The majority of participants in these studies reported that they would be averse to or have mixed feelings about interviewing an ED sufferer for a job, or in considering them as a dating partner [24-26,28,33]. However, some community participants have shown a more compassionate attitude, indicating that AN and BN are severe distressing conditions which warrant sympathy [22,23,32].

At the same time, evidence suggests that certain aspects of ED behaviour, namely, those related to weight loss and weight control, are seen as desirable or admirable by college and university students [24,28] as well as by friends and family of sufferers [29]. Further, there is a perception among university students that young people may be likely to imitate the disordered eating behaviours of individuals with AN or BN [28], highlighting body image concerns and admiration of the ‘thin ideal’ in this demographic. The weight-control strategies of EDs are also seen as desirable by individuals who have EDs or ED symptoms [24,34,35]. Thus, in addition to stigma, the desirability of ED behaviours may play a role in the increased prevalence of these behaviours.

Much less research has examined community attitudes towards individuals with Binge Eating Disorder (BED). Bannon et al. examined differences in undergraduate psychology students’ responses to a written scenario describing obese women with and without binge eating. The majority of students considered the obese woman with binge eating to be less attractive, less comfortable to be around, more likely to be blamed for her weight, and more difficult to treat, than the non-binge eating obese female [36]. Ebneter et al [35] examined stigma among university students towards people with a number of conditions, AN, BN, BED with obesity, obesity alone, and depression [37]. In contrast to the findings of Bannon and colleagues, individuals with obesity alone were most often blamed for their condition in this study [36,37]. Perceived lack of self-discipline was seen to be most likely among obese people, with or without BED, compared to the AN, BN and depression [37].

More generally, obesity stigma and discrimination have been found to be common and exist in a wide range of contexts including workplaces, health care, education, mass media and personal relationships [11,12]. Andreyeva et al. found that the prevalence of weight discrimination in the US population increased from 7% in 1995–1996 to 12% in 2004–2006 [38]. An increase in weight-based discrimination is important as those discriminated against are at risk of increased body dissatisfaction, poor self-esteem, binge eating and poor quality of life [7-15].

To our knowledge, no research has examined discrimination against obese BED and normal weight eating disorder (NWED) sufferers in a representative population sample. Understanding levels of community discrimination against ED sufferers of different weight status is important, as only behaviours and attitudes that are high prevalence are likely to make for effective targets in population health campaigns. The goal of the current study was to investigate perceived discrimination and positive attitudes toward three types of ED sufferers: a female sufferer of co-morbid obesity and BED; a female underweight sufferer of AN; and a male NWED sufferer. In addition, within each problem-type we considered associations between perceived discrimination and positive regard, and participants’ demographic and other characteristics, namely, their age, gender, level of education, body weight and ED features. To explore putative effects on help-seeking behaviour, we examined associations between perceptions of discrimination and perceptions of treatment difficulty.

It was hypothesised that there would be a relationship between positive regard and discrimination, such that as positive regard for a disorder increased, perceived discrimination would decrease. It was also expected that there would be higher levels of positive regard for the character with AN than for that the NWED character, given that the AN character was underweight. A third hypothesis was that perceived discrimination would be high across all three disorders: AN, NWED, and BED. Finally, it was hypothesised that participants at higher risk for EDs, namely females, obese participants, and participants regularly engaging in ED behaviours, would have higher regard for the weight loss behaviours of the female AN sufferer, and the shape control behaviours of the male NWED sufferer, than remaining participants.

## Methods

A large, cross-sectional, single-stage general population survey was conducted in the South Australian population, under the auspices of the South Australian Health Commission, in 2005. The survey questions for the present study were embedded in a larger survey assessing a range of health related and demographic questions. Face-to-face personal interviews were conducted by an independent survey research firm. Both metropolitan and rural households were surveyed. For the metropolitan sample, 386 “collectors’ districts” were selected from those used by the Australian Bureau of Statistics in the 2001 census. For the rural samples, all towns of 10,000 or more in population size and a selection of towns of at least 1,000 people were surveyed. The collectors’ districts were chosen according to their probability of selection proportional to size. Within each collector’s district, a starting point was randomly selected. From this starting point, using a predetermined process based on a “skip” pattern of every fourth household, 10 dwellings were chosen. Only one interview was conducted per household or dwelling, and, where more than one resident was aged over 15 years, the respondent was the person whose birthday was last. The sample was a non-replacement sample, and up to six separate visits were made to interview the person chosen to take part. Prior to commencing the interviews a pilot study of 50 interviews was conducted to validate the survey instrument, ensure participant understanding of interview questions and assess survey procedures. Following the completion of the interviews 10% of each interviewer’s work was selected at random. The respondents concerned were recontacted and a number of questions were asked of them to ensure they had been interviewed as reported.

Attitudes and beliefs concerning ED behaviours were assessed in the final section of the survey, in which a vignette of a fictional person suffering from an ED was presented, followed by a series of questions concerning the problem described. After the vignettes were read aloud to participants a written copy was provided to them, in order that they could reflect further on the vignette as the interview proceeded. Chosen households were allocated at random to one of three different versions of the survey, containing vignettes of underweight AN, atypical NWED and obese BED, respectively, so that approximately one third of participants responded to each vignette type. The vignettes were modelled on earlier vignettes used in ED population-based ED research to assess community beliefs regarding eating disorders [24,34,39]. The vignettes are provided in Appendix.

Perceived discrimination towards the person in the vignettes was assessed with the question “Do you think that Jenny (or Alison or Andrew) would be discriminated against by others in the community if they knew about her/his problems, for example, by an employer, a colleague, a family member, or by a health professional?”. In the obese BED vignette an additional question was asked to determine if such discrimination was perceived to be primarily ED-related or primarily weight-related.

Positive regard for the symptoms in the AN vignette was examined with the question “Have you ever thought that it might not be too bad to be like Jenny, given that she has been able to lose a lot of weight?”, whereas positive regard for the symptoms in the male NWED vignette was examined with the question “Have you ever thought that it might not be too bad to be like Andrew, given what he has been able to attain e.g. a good muscle tone and high level of exercise?”. A five point rating scale (‘never’ to ‘always’) was used for this purpose. The expression “it might not be too bad” is an Australian colloquial phrase which conveys a mildly positive feeling. Positive regard for the ED behaviours of the obese BED sufferer (i.e. binge eating) was not assessed as there was no rationale for this response.

Perceived difficulty of treatment for the respective conditions was assessed with the question “How difficult do you think Alison’s (of Jenny’s or Andrew’s) problem would be to treat?” A five-point rating scale (‘not difficult at all’, through to ‘extremely difficult’) was also used for this item.

Four ED features were assessed, namely, binge eating, purging, strict dieting or fasting, and extreme weight or shape concerns. Binge eating was described as an episode of “eating an unusually large amount of food in one go and at the time feeling that the eating was out of control, [that is you could not prevent yourself from overeating or that you could not stop eating once you had started]”. Participants were asked “over the last three months, how often have you overeaten in the way I have described” and were given four options ranging from ‘not at all’ through to ‘two or more times a week’. Purging was described as having “used laxatives, diuretics (water tablets), or made yourself sick, in order to control your shape or weight”. Strict dieting was described as “going on a very strict diet” or “eating hardly anything at all for a time”, for the purpose of weight or shape control. Current regular use of these behaviours was defined as the behaviour occurring at least weekly over the three months prior to the interview. The occurrence of extreme weight or shape concerns (‘undue influence of weight or shape on self-evaluation’) was assessed with the question “Has your weight and/or your shape influenced how you think about (judge) yourself as a person?”. A six-point response scale (‘not at all’, through to ‘extremely’) was used for this item. For the purpose of the current study, participants with a score of 4 (moderate influence) or more, were considered to have undue influence of shape or weight on self-evaluation. Body mass index (BMI; kg/m2) was calculated from self-reported weight and height. Obesity was classified as a BMI of 30.0 kg/m2 or above using the classification scheme outlined by the World Health Organization.

All participants gave informed consent. Ethics approval was granted by the South Australian Department of Health Ethics Committee.

### Statistical analysis

Data are presented as the percentage of participants choosing particular options for each question. Data were weighted by the inverse of the individual's probability of selection, then re-weighted to benchmarks for age, sex, and Local Government Area derived from the Australian Bureau of Statistics (Catalogue No 3204.4). The effects of obesity status, sex, age, educational status, and regular ED behaviours on levels of perceived discrimination and positive regard were examined by means of Chi-Square tests. Mann–Whitney U tests were employed to examine between-group differences in perceived discrimination and positive regard or treatment difficulty. In view of the multiple tests, a significance level of .01 was employed. All tests were conducted using the software SPSS version 22.

## Results

### Demographic characteristics of participants

From 4827 households selected for interview, interviews were completed with 3,047 individuals (63.1%). The demographic characteristics, weight status and ED features of participants are shown in Table 1.

**Table 1:** **Participant Demographic and Clinical Characteristics (n = 3047)**

	**Median (Interquartile Range)**
Age in years	49 (35–63)
BMI	25.3 (22.4- 28.8)
	**Percentage**
Obese (BMI ≥30)	19
Gender	
Female	58
Male	42
Age group	
15-34 years	33
35-54 years	36
55 years or more	31
Eating disorder behaviours	
Regular binge eating	6.8
Regular purging	1.8
Regular extreme dieting and fasting	4.2
Undue influence of weight and shape in self- evaluation as a person	18
Country of birth	
Australia	76
UK/Ireland	11
Other Europe	8
Asian	3
Other	2
Australian first nations	4
Marital status	
Married	50
Never married	20
Defacto	7
Divorced	13
Widowed	10
Highest level of education	
Still at school	4
Left school at 15 years or less	16
Left school after 15 years	25
Left school but still studying	4
Trade qualification	13
Certificate/diploma 1 year or less	12
Certificate/diploma more than 1 year	12
Bachelor degree or more	16

Of the total sample, 1030 participants (34%) were selected to answer the questions pertaining to the co-morbid obese/BED vignette, 1033 (34%) those pertaining to the male NWED vignette, and 983 (32%) those pertaining the AN vignette. The proportion of male and female participants differed across vignettes (obese BED: female = 61%; NWED: female = 54%; AN: female = 58%) (χ^2^ = 10.52, df = 2, p = 0.005). There were no other differences between groups with respect to demographic characteristics, weight status or ED features.

### Perceived discrimination and positive regard

Responses to the items assessing perceived discrimination and positive regard for each vignette are summarised in Table 2. Among participants who believed that the obese BED sufferer would be discriminated against, most (84%) believed this would be due to weight problems, 14% thought it would be due to eating problems and the 3% reported that they did not know.

**Table 2:** **Community perceived discrimination and positive regard toward a female anorexia nervosa (AN) sufferer, male normal weight eating disorder sufferer (NWED) and female sufferer of comorbid obesity and binge eating disorder (BED)**

	**Female AN Vignette (total n = 983) %**	**Male NWED (total n = 1033) %**	**Female Obese/BED Vignette (Total n = 1030) %**
Perceived Discrimination would occur against person in vignette			
Yes	48	35	66
No	45	60	28
Unsure	7	6	5
Positive regard: thought it would not be too bad to be like the person in vignette given weight loss (in AN vignette) or good muscle tone and exercise (in NWED vignette)			N/A
Never thought it	85	63	
Rarely thought it	7	10	
Occasionally thought it	7	20	
Often or always thought it	1.8	7.5	

### Influence of participant characteristics on perceived discrimination and positive regard

There were no significant differences between obese and non-obese participants, those with or without a bachelor degree or higher education, or those with and without ED features, in terms of perceived discrimination towards the BED character. Females were less likely than males (63% vs 71%) to think that the obese BED character would be discriminated against (χ^2^ = 10.682, df = 2, p = 0.005). Younger participants (age 15–34 years) were less likely (58%) than those aged 35–54 years (71%) or those aged more than 55 (67%) to believe the obese BED character would be discriminated against (χ^2^ = 21.713, df = 4, p < 0.001). The proportion of participants who believed that there would be discrimination, whether for weight or eating problems, did not significantly differ by participants’ weight (obese/non-obese) status (χ^2^ = 1.63, df = 1, p = 0.20).

In order to examine the influence of weight status and demographic features on responses to the positive regard item, these responses were recoded into two categories: those who had never or rarely had these thoughts and those who had occasionally, often or always had these thoughts. For the AN vignette, participant weight status, ED behaviours and level of education (bachelor’s degree, no bachelor’s degree) were not associated with perceived discrimination. Younger participants (15–34 years) were less likely (44%) than those aged 35–54 years (50%) or those aged more than 55 (48%) to believe that discrimination towards the AN character would be likely (χ^2^ = 13.223, df = 4, p = 0.010). Sex, age, education status, and the occurrence of purging did not significantly influence positive regard for the AN character. However, obese participants were significantly more likely (n = 26, 15%) than those who were not obese (n = 53, 7%) to report positive regard for AN symptoms (χ^2^ = 9.446, df = 1, p = 0.002). Participants reporting regular fasting were significantly more likely (n = 10, 23%) to have high positive regard for the AN character than those who did not report fasting (n = 74, 8%) (χ^2^ = 12.531, df = 2, p < 0.002). Participants reporting regular (> once a week) binge eating were significantly more likely (n = 20, 32%) than those who did not have regular binge eating (n = 64, 7%) to report a high positive regard for the AN character (χ^2^ = 47.612, df = 1, p < 0.001). Finally, participants with undue influence of shape or weight on self-evaluation were significantly more likely (n = 28, 17%) than participants who did not have this feature (n = 56, 7%) to report high positive regard for the AN character (χ^2^ = 17.350, df = 1, p < 0.001).

For the male NWED vignette, there were no significant differences between obese and non-obese participants, participants with or without ED features, male and female participants, and participants with or without a Bachelor’s degree, in responses to the item assessing perceived discrimination. However, participants aged 15–34 years and 35–54 years were less likely (29% and 32% respectively) to perceive discrimination than those aged 55 years or more (41%) (χ^2^ = 26.068, df = 4, p < 0.001). Sex, educational attainment, and the presence of purging behaviours did not significantly influence positive regard for NWED symptoms. However, younger participants (15–34 years, 33%) and those aged 35–54 years (33%), were more likely than participants aged 55 years or more (18%) to have high regard for the NWED character (χ^2^ = 28.075, df = 2, p < 0.001). Further, obese participants (43%) were more likely than non-obese participants (24%) to have high regard for the NWED character (χ^2^ = 25.171, df = 1, p < 0.0001). Participants engaging in regular fasting (54%) were more likely to have high regard for the NWED character than participants not regularly engaging in fasting (26%) (χ^2^ = 16.2750, df = 2, p <0.0001) and participants who reported regular binge eating (54%), were more likely to have high regard for the NWED character then those who did not (25%) (χ^2^ = 26.319, df = 1, p < 0.0001). Finally, participants with undue influence of shape or weight on self-evaluation were significantly more likely to have high regard for the NWED symptoms (47%) than those who did not have this feature (23%) (χ^2^ = 54.712, df = 1, p < 0.0001).

### Association between perceived discrimination and positive regard for ED behaviours

Levels of positive regard for the weight-reducing behaviours of the AN character did not differ between participants who perceived discrimination towards this character (n = 463) and those who did not (n = 463) (median levels in both groups = ‘never’, IQR ‘never’-‘never’; Mann–Whitney U test p = 0.41). Levels of positive regard for the muscle tone enhancing and exercise behaviours of the person in the NWED vignette were significantly lower in the 346 participants who perceived discrimination against him (median regard ‘never’, IQ range ‘never’-‘rarely’), compared to the 640 who did not perceive discrimination against him (median regard ‘never’, IQ range ‘never’-‘occasionally’; Mann–Whitney U test p < 0.02).

### Association between perceived discrimination and treatment difficulty

Perceived treatment difficultly for the AN character was higher among participants who perceived discrimination towards this character (median = ‘moderately’, IQR = ‘moderately’ to ‘very difficult’) than those who did not (median level ‘moderately’, IQR = ‘a little’ to ‘very difficult’; p < 0.001). Perceived treatment difficulty for the NWED character were higher among participants who perceived discrimination against this character (median = ‘moderately difficult’, IQR = ‘a little’ to ‘moderately’) than those who did not (median = ‘a little’, IQR = ‘not at all difficult’ to ‘moderately difficult’; p < 0.001). Perceived treatment difficulty for the BED character were also higher among participants who perceived discrimination against this character (median = ‘moderately difficult’, IQR = ‘moderately-‘very difficult’) than those who did not (median = ‘moderately’, IQR = ‘a little’ to ‘very difficult’; p < 0.001).

## Discussion

In this general population study, the majority of participants thought that discrimination against an obese individual with BED would occur, just under half believed that discrimination towards an underweight woman with AN would occur, and a third thought that a male of normal weight with an ED would experience discrimination. Perceived discrimination across different types of ED is consistent with previous research [22-29,36,37]. Additionally, when forced to choose the main reason for discrimination, the majority of participants believed that discrimination towards the obese BED character would be weight-, rather than ED-related. This finding is consistent with findings from studies of obesity stigma and discrimination [11,12,37,38] indicating that weight stigma may make obese individuals with EDs a particularly vulnerable subgroup.

In the current study, a small number of participants had a favourable regard towards the AN character and more (around a third) had favourable regard toward the NWED character. This is consistent with previous studies suggesting that the public views certain ED features to be desirable [24,28,29]. Further, obesity and the presence of ED symptoms appears to increase the likelihood of having positive regard for individuals with EDs. Thus, many of the participants with a favourable regard for ED features had ED symptoms and/or were obese and are, therefore, at increased risk of developing an ED. Interventions for individuals at risk of EDs have been successful in reducing the incidence of ED symptoms [40]. Thus, early interventions for these individuals assisting them to adopt healthy body image and eating behaviours may assist in stemming the increase in EDs and mental health problems among obese individuals.

The results of this study support calls for an integrated approach to ED and obesity prevention [41,42], and also calls to stop ‘fat shaming’ in obesity campaigns [12,43]. Recent universal ED prevention programs have focused on body image, in light of evidence from some early studies suggesting potential iatrogenic effects in programs directly discussing ED behaviours [40]. Obesity prevention trials continue to focus on changing individuals’ diet and exercise behaviours, despite lack of evidence for the effectiveness of this approach [44]. Research into the reduction of obesity stigma is in its infancy, but has shown promising results [45]. Interventions that show promise have used anti-stigma films that challenge negative stereotypes, improve understanding of the complex aetiology of obesity and obesity discrimination [46], employed used cognitive dissonance principles [47], or combined these strategies [48].

To our knowledge reducing obesity stigma has not yet been a target of universal ED or obesity prevention efforts. Given the high perceived levels of weight discrimination in the present study, and the link between obesity stigma and poor body image, depression, binge eating, and reduced physical activity [7-15], it may be worth investigating if obesity stigma is amenable to change at a population level and therefore if it can influence rates of body dissatisfaction, depression, binge eating and physical activity. Given the high levels of discrimination against obese individuals with BED in particular, and the increasingly high prevalence of this comorbidity, reducing stigma towards these individuals should be a priority. In this regard, it may be beneficial to highlight the high costs in terms of quality of life impairment associated with comorbid ED behaviour while also providing information about help seeking and treatment options. The current findings also indicate the need to address societal influences likely to be conducive to disordered eating and obesity, for example, public planning, media influences, and the cost of healthful foods, rather than placing the onus on individuals to change their eating behaviour, the latter approach potentially being conducive to greater self-stigma and unhealthy eating and weight-control behaviours.

The finding that perceived discrimination was related to perceived treatment difficulty may help to explain the observation, in previous research, of an association between stigma and low help seeking [30-32]. The implications of the finding, also observed in previous studies, that a proportion of the community has favourable regard for ED symptoms, are unclear. A concern requiring further research is that favourable regard may reinforce ED behaviour in others and, perhaps, increase individuals’ own risk of ED behaviour. All three factors, discrimination, perceived treatment difficulty and positive regard, may act together as a barrier to individuals’ receiving professional treatment when this is needed.

Limitations of the current research should be recognised. First, no information was obtained concerning what form the hypothesized discrimination might take. Although various examples (such as discrimination by an employer, a colleague, a family member, or by a health professional) were given, participants did not have the opportunity to indicate which of these or other forms of discrimination might be more or less likely. Similarly, stigma was not assessed on a personal level, for example, how comfortable would the participant be socialising or working with a sufferer with the problem described, or if a similar problem had caused the participant to be discriminated against and the nature of that discrimination. Since there was no obese only vignette, whether and how perceived discrimination might differ for obese individuals who have and who do not have BED could not be determined. It should also be noted that BMI was derived from self-reported rather than measured height and weight. It is possible that differences in the sex of the vignette characters and/or the use of a male character in the NWED vignette may account, in part, for findings relating to perceived discrimination and positive regard. It also is possible that the sex and physical attributes of the interviewer may have been conducive to a social desirability bias in responses. This possibility could not be examined. Complexity in how the different genders, age, professions of the vignettes may have also played an unknown role in people’s responses. Further, we cannot determine the degree that participant responses to positive regard were based solely on the aspect in the question and not on other aspects of the vignette. Strengths of the current research include the recruitment of a large, representative general population sample, the inclusion of 3 different vignettes, and the assessment of participants’ ED behaviour in addition to their body weight and demographic characteristics.

## Conclusion

In a large, general population sample we found high levels of perceived discrimination towards an obese female with BED and an underweight female with AN, whilst a male NWED sufferer was seen to be less likely to experience discrimination. A minority of the community, especially obese persons, found the weight-reducing and body-image-enhancing aspects of people with EDs desirable. Public health interventions targeting ED or obesity need to be mindful not to contribute to the positive regard for ED behaviours or to the high levels of stigma towards obese individuals and those with EDs. The potential benefits of reducing obesity stigma at the population level warrants investigation.

## Appendix

### Vignette 1

Jenny is a 28 year old “stay at home Mum”. She has 3 young children and has recently stopped breastfeeding. Despite major efforts to lose weight in the last five years with a number of diets, she has not had much success until recently. In the last 6 months Jenny has started jogging every night, when her husband arrives home to look after the kids. If she ever misses a night she feels guilty and upset and jogs twice as far the next day. In the last few months Jenny has cut back on her food intake while her husband is at work, she often skips breakfast and only has a small salad for lunch. Jenny has also started secretly vomiting after her husband cooks high fat dinners for the family. Jenny thinks she is fat and worthless; although she is enjoying compliments she has obtained from her husband regarding weight loss (about 10 kg). Jenny is 168 cm tall and has a present weight of 44 kg (BMI =15.6). She looks thinner than most ‘supermodels’.

### Vignette 2

Andrew is a 26 year old male who works in the meat works. Andrew is normal weight with good muscle tone, but feels that he has a “pot belly”. Andrew is very worried about his looks and wants to bulk up his muscles and lose fat. Andrew’s job involves a lot of physical labour but he does not count this as exercise. Every night Andrew spends an hour and a half in the gym lifting weights, and on his day off he goes for a 15 km run. Andrew has recently started replacing his dinner meal with a high protein sports drink. He also tries to eat high protein foods through the rest of the day. He sometimes (about twice a week) has uncontrolled eating ‘binges’ where he eats e.g. half a loaf of bread in the late afternoon. Andrew does not have many friends and feels that if he changes his shape he will be more attractive and a better person.

### Vignette 3

Alison is a 32 year-old secretary working at a solicitor’s office. Alison has been overweight since she was an adolescent but in recent years this has increased to where she is now a size 18 and has been told she has ‘severe obesity’. Over the years Alison has tried a number of diet and healthy eating plans; however she has never stayed with the recommendations for very long. Alison lives by herself and often feels lonely; to counteract these feelings Alison likes to ‘treat’ herself with luxurious foods such as chocolate and cheesecake. Alison’s diet is regular with 3 meals a day and it contains a wide variety of foods. When Alison gets home from work she often goes to the fridge for a small snack, however Alison finds that after eating the snack she is unable to stop eating and continues to eat a large amount of food. She may eat for example an apple, a slice of cheesecake, 5 biscuits, a jam sandwich and three glasses of milk. Later in the evening she will eat dinner and sometimes she loses control with this also and eats the extra helping that she was planning to save for the next day. Alison feels guilt and sadness after she has eaten like this and despises the shape of her body. Alison has never told anyone about the way she feels or the way she loses control of her eating. She has often thought about different ways to control her weight (e.g. exercise or laxatives) but has never done them.
